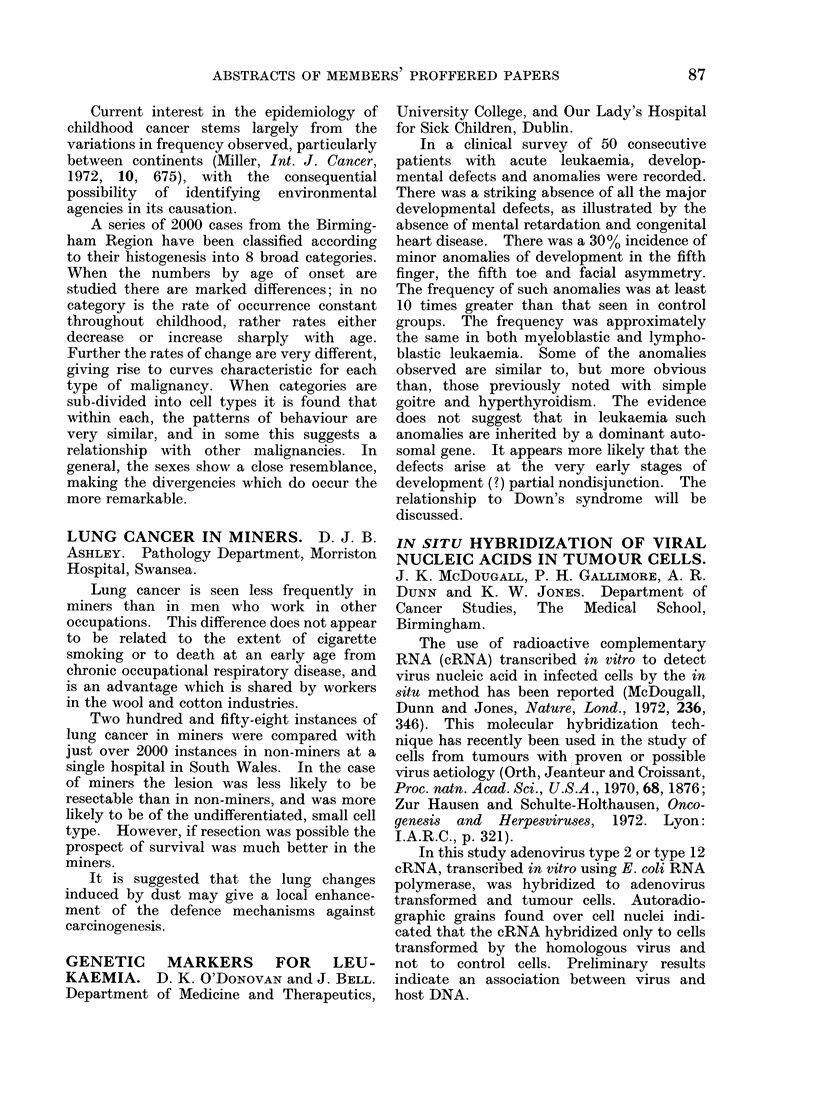# In situ hybridization of viral nucleic acids in tumour cells.

**DOI:** 10.1038/bjc.1973.113

**Published:** 1973-07

**Authors:** J. K. McDougall, P. H. Gallimore, A. R. Dunn, K. W. Jones


***IN SITU* HYBRIDIZATION OF VIRAL NUCLEIC ACIDS IN TUMOUR CELLS.** J. K. McDougall, P. H. Gallimore, A. R. Dunn and K. W. Jones. Department of Cancer Studies, The Medical School, Birmingham.

The use of radioactive complementary RNA (cRNA) transcribed *in vitro* to detect virus nucleic acid in infected cells by the *in situ* method has been reported (McDougall, Dunn and Jones, *Nature, Lond.*, 1972, **236**, 346). This molecular hybridization technique has recently been used in the study of cells from tumours with proven or possible virus aetiology (Orth, Jeanteur and Croissant, *Proc. natn. Acad. Sci., U.S.A.*, 1970, **68**, 1876; Zur Hausen and Schulte-Holthausen, *Oncogenesis and Herpesviruses*, 1972. Lyon: I.A.R.C., p. 321).

In this study adenovirus type 2 or type 12 cRNA, transcribed *in vitro* using *E. coli* RNA polymerase, was hybridized to adenovirus transformed and tumour cells. Autoradiographic grains found over cell nuclei indicated that the cRNA hybridized only to cells transformed by the homologous virus and not to control cells. Preliminary results indicate an association between virus and host DNA.